# Brain-Implantable Multifunctional Probe for Simultaneous Detection of Glutamate and GABA Neurotransmitters: Optimization and In Vivo Studies

**DOI:** 10.3390/mi13071008

**Published:** 2022-06-26

**Authors:** Sanjeev Billa, Yaswanthi Yanamadala, Imran Hossain, Shabnam Siddiqui, Nicolaie Moldovan, Teresa A. Murray, Prabhu U. Arumugam

**Affiliations:** 1 Institute for Micromanufacturing (IfM), Louisiana Tech University, Ruston, LA 71272, USA; sbi005@latech.edu (S.B.); buet.imran@gmail.com (I.H.); 2 Center for Biomedical Engineering and Rehabilitation Science (CBERS), Louisiana Tech University, Ruston, LA 71272, USA; yya012@latech.edu (Y.Y.); tmurray@latech.edu (T.A.M.); 3 Department of Chemistry and Physics, Louisiana State University Shreveport, Shreveport, LA 71115, USA; shabnam.siddiqui@lsus.edu; 4 Alcorix Co., Plainfield, IL 60544, USA; moldovan@alcorix.com

**Keywords:** biosensor, glutamate, GABA, real-time sensor, in-situ calibration, in vivo recording

## Abstract

Imbalances in levels of glutamate (GLU) and gamma-aminobutyric acid (GABA) and their sub-second signaling dynamics occur in several brain disorders including traumatic brain injury, epilepsy, and Alzheimer’s disease. The present work reports on the optimization and in vivo testing of a silicon (Si) multifunctional biosensor probe for sub-second simultaneous real-time detection of GLU and GABA. The Si probe features four surface-functionalized platinum ultramicroelectrodes (UMEs) for detection of GLU and GABA, a sentinel site, and integrated microfluidics for in-situ calibration. Optimal enzyme concentrations, size-exclusion phenylenediamine layer and micro spotting conditions were systematically investigated. The measured GLU sensitivity for the GLU and GABA sites were as high as 219 ± 8 nA μM^−1^ cm^−2^ (*n* = 3). The measured GABA sensitivity was as high as 10 ± 1 nA μM^−1^ cm^−2^ (*n* = 3). Baseline recordings (*n* = 18) in live rats demonstrated a useful probe life of at least 11 days with GLU and GABA concentrations changing at the levels of 100′s and 1000′s of μM and with expected periodic bursts or fluctuations during walking, teeth grinding and other activities and with a clear difference in the peak amplitude of the sensor fluctuations between rest (low) and activity (higher), or when the rat was surprised (a reaction with no movement). Importantly, the probe could improve methods for large-scale monitoring of neurochemical activity and network function in disease and injury, in live rodent brain.

## 1. Introduction

Several brain disorders are linked to imbalances in glutamate (GLU) and gamma-aminobutyric acid (GABA) homeostasis [1,2,3,4,5]. GABA is the major inhibitory neurotransmitter (NT), and GLU is the major excitatory neurotransmitter (NT) [6,7,8]. Dysregulation of GLU and GABA occurs in several neurological disorders, including epilepsy, dementia (a disorder that will affect 130 million worldwide by 2050), Parkinson’s disease, Schizophrenia, drug use and addiction [7,8,9,10]. A fundamental understanding of GLU and GABA dynamics in various brain regions would likely lead to a better understanding of human brain function and to the development of new and more effective treatments (e.g., DBS, neuromodulation technologies).

Even though considerable progress has been made in understanding the basic relationships between human behavior and brain function and between symptoms and brain pathology, the complexity of brain physiology and neurochemistry clearly speaks to the need for further discovery and technology development in this field. Advances in many approaches, such as genomics, proteomics, imaging, and electrical monitoring, have contributed greatly towards understanding the human brain and treating neuropathologies [11,12]. However, assessment of dynamic NT events has been relatively neglected in recent years, in large measure since the sensing techniques are cumbersome compared to imaging (e.g., fMRI). Development of advanced NT sensors that can measure the fluctuating patterns of NT activity within neural circuits across time and space and illuminate the interplays with electrical activity, which creates unique cognitive and behavioral capabilities, is crucial. The current gold standard method to detect NTs is microdialysis [13,14,15,16,17,18,19]. Unfortunately, microdialysis is an offline technique that suffers from poor spatiotemporal resolution and inability to measure NT dynamics at the circuit level, continuously in real time [14,15]. In contrast, electrochemical biosensors are easy to miniaturize [13,14,15,16,20,21] and are essential for chronic, real-time, in vivo NT recordings. Electrochemical biosensors patterned in a microarray format could enable researchers to evaluate the role of several key NTs on behavioral events within and across brain areas and develop a deeper understanding of the NT activity of neuronal circuits due to different modulation strategies, a central goal of the United States’ Brain Research through Advancing Innovative Neurotechnologies (BRAIN) Initiative. Electrochemical detection of non-electroactive NTs, such as GLU and GABA, relies on enzymatic reactions to generate electrochemically active molecules such as hydrogen peroxide (H_2_O_2_) [22,23,24]. Platinum (Pt) microelectrodes micropatterned on substrate materials (e.g., ceramic, silicon) in a microarray format are generally used to oxidize H_2_O_2_ thus generating a detection current which is proportional to NT concentration. Historically, detection of GABA necessitated the application of pre-reactors to mitigate the effects of interferents and the application of an enzyme substrate [25,26,27]. Adding exogenous compounds is not practical for chronic, deep brain recordings in awake rodents. Our novel approach utilized two closely spaced Pt microelectrodes modified with glutamate oxidase (GOx) and GOx + GABASE to measure GLU and GABA simultaneously without the need for any externally applied substrate [28]. The GLU present in the brain environment was used as an in-situ source for the generation of α-ketoglutarate, a substrate for GABA oxidation. Detailed preliminary characterization of this simultaneous GLU and GABA detection principle was published in [28,29] Changes in GLU and GABA levels related to various behaviors were monitored by simultaneously measuring the H_2_O_2_ currents generated from the two microbiosensors. However, some shortcomings exist in calculating concentration and for chronic use. For example, the conversion of NT signals to concentration was likely inaccurate since conversion depends on calibrations performed in vitro. In addition, the relatively large, shank-shaped microarrays currently available for coating with enzymes are, bulky, and not suitable for chronic use. 

**Figure 1:**
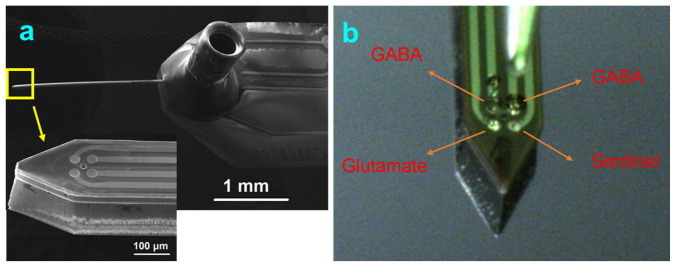
(**a**) SEM images of the Si probe with microfluidic connector. The probe is 200 μm × 200 μm × 10 mm. The four Pt UMEs are 25 μm in diameter. The spacing between the UMEs is 50 μm. The UMEs surrounds the microfluidic port located at the center. (**b**) Optical image of the probe tip showing the surface modification of UMEs with enzyme and matrix coatings.

To overcome the above technical challenges, in prior work, authors microfabricated and characterized a multifunctional, chronically implantable silicon (Si) electrochemical biosensor probe (200 μm × 200 μm × 10 mm, Figure 1a) with in-situ calibration capability that allowed GLU and GABA detection with sub-second temporal resolution in the 100 ms range. The Si probe consisted of four 25-μm diameter Pt ultra-microelectrode array (UMEs) with adequate mechanical, electrical, electrochemical, and microfluidic characteristics for in vivo implantation and recordings [30]. A microfluidic channel was integrated into the Si probe as an on-demand, in-situ calibrator (ODIC) for a more accurate calibration for long-term in vivo recordings. The four UMEs within the Si probe are designed to detect GLU and GABA simultaneously with a built-in sentinel (control) site to subtract signals from interferent molecules. The GLU site is coated with glutamate oxidase (GOx). The GABA sites are coated with GABASE and GOx enzymes. For the GLU site, GOx facilitated the breakdown of non-electrically active GLU into -ketoglutarate (α-keto), NH_3_ and hydrogen peroxide (H_2_O_2_). A +0.7 V bias on the underlying Pt microelectrode oxidized H_2_O_2_ molecules, releasing two electrons per H_2_O_2_ molecule which produced a measurable current that was proportional to the NT concentration. This current was converted into GLU concentration based on pre-calibration values. For the GABA site, the combined current generated from GLU and GABA was recorded. The current from an adjacent GOx-coated electrode was subtracted from this signal to derive the GABA current [28,29]. The GOx enzyme on the GABA-GLU biosensor produced enough α-keto to facilitate the conversion of GABA to H_2_O_2_ by GABASE. The sentinel site was coated identically to the other sites, except that no enzymes were incorporated into the coating. The sentinel enabled subtraction of signals from electrically-active interferent molecules, such as ascorbic acid (AA). In addition, a size-exclusion layer of m-phenylenediamine (mPD) was applied over all probe sites to reject larger sized molecules, including most AA molecules, which mitigated the effects of interferent molecules and further improved selectivity.

In this work, the process variables were optimized to achieve further improvements in the sensor performance of the authors prior work [30] and validated the probe’s performance chronically in live rats. Here, the authors (1) optimized the concentrations of the two enzymes, GOx and GABASE, the coating of the mPD layer and the micro spotting of the two enzymes to achieve the highest GLU and GABA sensitivity and selectivity, (2) characterized the ODIC (built-in microfluidic channel) and (3) evaluated the probe in a live rat in terms of % signal loss over 11 days and usefulness in correlating rat behaviors with fluctuations in GLU and GABA concentration. 

## 2. Materials and Methods

### 2.1. Design Considerations and Microfabrication

The general design of the probe fulfills some of the challenging requirements such as robustness during probe insertion; small profile to avoid causing significant tissue damage; adequate electrode size for good NT sensitivity while being small enough for low-volume local detection; a large-enough microchannel to facilitate efficient delivery of chemicals and drugs. These are detailed in [30]. A finished Si probe is shown in Figure 1a. Production details for the Si probe with integrated UMEs and microfluidic channel are described in [30]. Briefly, the Si probes were fabricated on 100-mm-diameter Si (100) prime grade wafers with a thickness of 200 µm which was the thickness of the finished probes. Since the thickness of the A small probe size is essential for minimizing brain tissue damage at insertion; thus, 200 µm was a trade-off between having a small profile and maintaining robustness of the wafers during processing. However, the results obtained with fabricating and using these probes suggest that further reduction of the probe size is possible.

### 2.2. Electrochemical Characterization 

The Pt UMEs were cleaned electrochemically by first submerging them in 0.05 M H_2_SO_4_ electrolyte solution (Sigma Aldrich, St. Louis, MO, USA) and cycling the voltage Cyclic Voltammetry technique, CV) between −0.3 V and +1.0 V using a 20 mV/s scan rate for 5 cycles (details in references [29,31]). Electrochemical calibration experiments were performed using an Autolab potentiostat (PGSTAT 302N, Metrohm Autolab, Riverview, FL, USA) in a 3-electrode configuration with a saturated calomel reference electrode (SCE, Fischer Scientific, Waltham, MA, USA), a Pt coil (Alfa Aesar, Haverhill, MA, USA) used as the counter electrode, and the Pt UME used as the working electrode, and. All measurements were performed in freshly prepared solutions that were purged with nitrogen for 5 min prior to use. Chronoamperometry was performed using +0.7 V vs. an Ag/AgCl reference/counter electrode that was implanted next to the silicon probe in a 2-electrode setup for chronic in vivo recording. The Ag/AgCl reference electrodes was prepared in-house by coating the AgCl onto a 200 μm Ag wire (A-M Systems, Sequim, WA, USA) using a 5 M NaCl in 1 M HCl at 9 V for 1200 s using chronoamperometry technique.

### 2.3. Chemicals and Enzyme Coating

L-glutamate oxidase (YMS-80049) was purchased from Cosmo Bio USA (Carslbad, CA, USA). GABASE (G7509), sulfuric acid (CAS. 7664-93-9), hydrogen peroxide (CAS. 7722-84-1), potassium chloride (CAS. 7447-40-7), L-glutamic acid monosodium hydrate salt (CAS. 142-47-2), gamma-aminobutyric acid (CAS. 56-12-2), potassium hexacyanoferrate (III) (CAS. 13746-66-2), potassium hexacyanoferrate (II) trihydrate (CAS. 14459-95-1), glutaraldehyde solution (CAS. 111-30-8,67-56-1), and bovine serum albumin (CAS. 9048-46-8) were purchased from Sigma Aldrich (St. Louis, MO, USA). Chemicals were used as received.

Enzyme coatings were previously described in detail in [28,30]. Briefly, a fresh enzyme matrix solution was prepared containing bovine serum albumin (BSA, 1%) and a crosslinker, glutaraldehyde (0.125%), in DI water. This crosslinking method retained enzyme structure and maintained enzyme functionality. To optimize the GLU microbiosensor, varying concentrations of GOx (0.1 to 0.8 U/μL) were mixed with the BSA-glutaraldehyde solution and tested. To optimize the GABA microbiosensor, varying concentrations of GOx (0.1 to 0.8 U/μL) and GABASE (0.1 to 0.8 U/μL) were tested [28,30]. The enzyme solutions were micro spotted onto the Pt UMEs using a Sonoplot Microplotter Proto. Authors systematically studied the effect of spraying strength, spraying time, and glass pipette size (5, 10, 20 µm) on spot size and spot repeatability. The spraying strength scales with the voltage applied to the piezoelectric transducer, which causes fluid to dispense via ultrasonic vibrations. The spraying time is the duration of the applied voltage. The 10 µm pipettes were ideal when lower dispensing voltage and time were used. This led to smaller droplet sizes ideal for the Pt UMEs (Figure 1b). About 50 droplets of GOx enzyme can be dispensed consistently using the 10 μm pipette as shown in Supplementary Material Figure S2. The 10 µm pipette was employed to dispense 1 to 2 drops (∼0.02 μL/drop) of the respective solution on to the UME sites. A new pipette was used for each new spotting of an enzyme solution to avoid clogging and cross contamination. The enzyme dispense volume was carefully controlled so that it did not overflow onto the neighboring UME sites. The coated devices were stored at room temperature, in the dark for 48 h to complete cross-linking of the coating [28,29]. Once cross-linked, a size-exclusion layer of mPD was electrochemically applied over the coated UMEs. This conferred selectivity by blocking the diffusion of interferent molecules, primarily AA, that are present in concentrations up to mM range. The mPD solution (10 mM) was prepared in 1 M NaCl and then purged with nitrogen for 30 min prior to use. The mPD was applied by cycling voltage across the UMEs between +0.2 V and +0.8 V at two scan rates (5 mV/s and 50 mV/s) for 5 or 10 cycles (Supplementary Information Figure S4).

### 2.4. Amperometry Calibration and Detection of NTs

An eight-channel FAST-16mkIII^®^ potentiostat with a 2-electrode configuration, with an Ag/AgCl reference electrode was used (Quanteon LLC, Nicholasville, KY, USA) for calibration and recording. Calibrations were conducted using amperometry with +0.7 V across the Pt UME electrodes in 50 mL 1X phosphate buffered saline solution (PBS) in a glass beaker (details of the sensing mechanism and calibration are described in Supplementary Information Figure S1) and in reference [31]. Measurements were repeated (*n* = 6) and the data were analyzed using FAST analysis^®^ software. Sensitivity toward the GABA or GLU was defined as the change in current per unit of added analyte, which is the slope of the calibration curves (pA/µM). To calculate the sensitivity per electrode area (SS, nAµM^–1^ cm^–2^), the slope was divided by the area (8.4 × 10^−6^ cm^2^) of a Pt UME. One-way analysis of variance (ANOVA) was performed to compare differences between coating conditions. Values on plots are shown as mean ± SEM (Standard Error of the Mean). Significance is defined as *p* < 0.05.

### 2.5. Probe Implantation and In Vivo Recording

Male Sprague–Dawley rats were provided with food and water ad libitum and housed on a 12 h light/dark cycle. All procedures were approved by the Louisiana Tech University Institutional Care and Use Committee and were in accordance with the Guide for the Care and Use of Laboratory Animals.

The shaft, proximal to the connection to the PBP board, was coated with a thin layer of dental acrylic (Ortho-Jet BCA, Lang Dental Manufacturing Company, Wheeling, IL, USA) to prevent breakage of the Si shaft at the PCB board connection during implantation [29]. Rats were briefly anesthetized with 4–5% isoflurane prior to administering an intramuscular injection of ketamine HCl (75 mg/kg) and dexmedetomidine HCl (0.25 mg/kg). A 1.59-mm diameter craniotomy was made 4.6 mm posterior to Bregma, 3 mm lateral to the midline over the right hemisphere using aseptic surgical techniques. Three stainless-steel anchor screws (Stoelting Co., Wood Dale, IL, USA), 1.25 mm in dimeter, were installed into the skull along the outer edge of the scalp incision to anchor the implant. The PCB board of the probe was affixed to an arm on a stereotaxic frame, positioned and then lowered at 100-μm per min to a depth of 2.5 mm. Dental acrylic was applied to the probe and screws to secure the probe to the skull. An analgesic, antibiotic, and anti-inflammatory powder (Neo-Predef, Zoetis, Florham Park, NJ, USA) was applied. Next, Atipamezole hydrochloride (1 mg/kg) was administered intramuscularly to bring the rat out of anesthesia. Rats were monitored until they moved around the cage. They were housed individually after surgery.

Prior to recording, rats were acclimated to the room for 20 min in a standard, clear plastic housing cage with corn cob bedding, a water bottle and several pieces of chow. To avoid stress associated with connecting the implanted microbiosensor to the recording system, each rat was briefly anesthetized using 5% isoflurane before attachment. For recording, the rat was returned to its cage with the cover removed. For recording NT signals, a +0.7 V bias voltage was applied to the biosensors to facilitate oxidation of H_2_O_2_. The FAST system acquired current data at 1000 Hz which were stored as csv files for offline analysis. The first recording was performed a few hours after surgery. Subsequent recordings were acquired up to 11 days (average 4.5 recordings/rat). Normal behaviors were entered into a log with the recording time for offline analysis.

## 3. Results and Discussions

### 3.1. Electrochemical Characterization

#### 3.1.1. H_2_O_2_ Calibration

**Figure 2:**
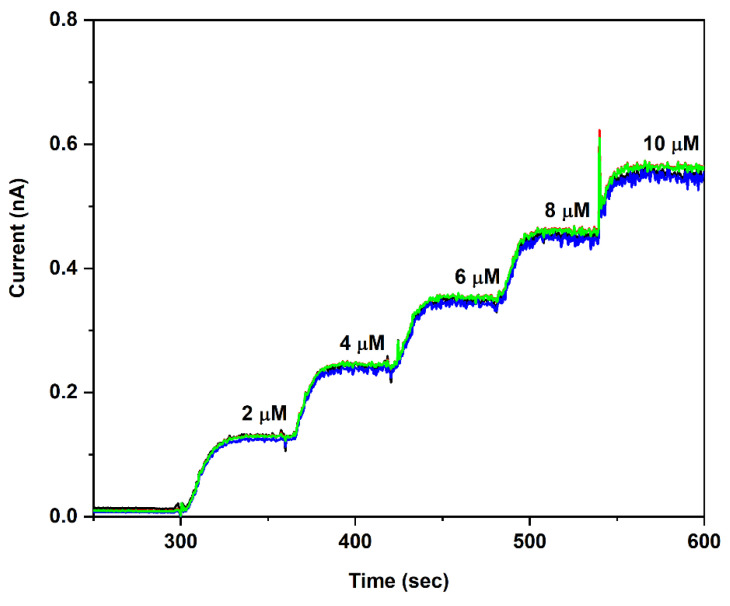
Overlay of amperometry calibration curves recorded from the four Pt UMEs within a Si probe. The calibration data of the four UMEs 1 to 4 are shown in black, red, blue, and green curves. The CV cleaned UMEs were biased at + 0.7 V vs. Ag/AgCl reference. The solution was stirred at 200 rpm and maintained at 37 °C.

The Pt UMEs were cleaned using CV to increase their sensitivity toward H_2_O_2_ and improve their biosensor performance (as detailed in reference [30]). They were then calibrated to measure their sensitivity towards H_2_O_2_. (This is the electroactive product of the GOx- and GABASE-mediated reactions [32,33,34]. Sensitivity values for H_2_O_2_ were on the order of 6000 ± 100 nA μM^−1^ cm^−2^ (*n* = 6) (Figure 2), which could be due to an increase in electrocatalytic sites after cleaning, as reported in [30].

#### 3.1.2. Effect of GOx and GABASE Concentrations on NT Sensitivity

**Figure 3:**
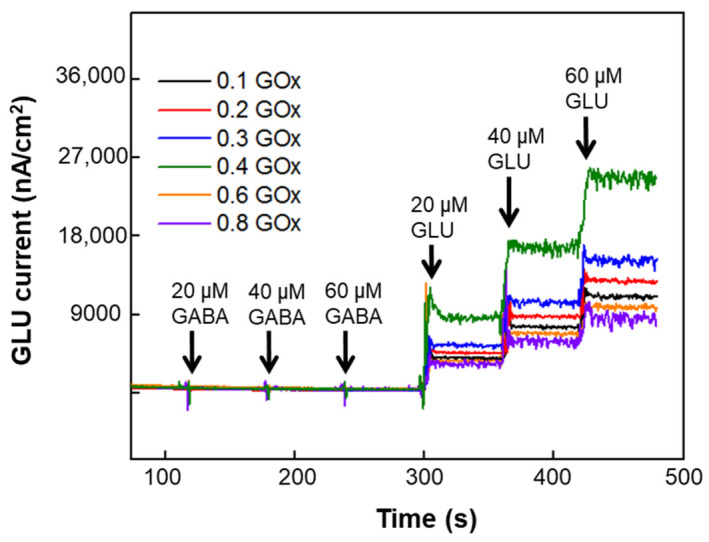
In vitro calibration of GLU microbiosensors at 0.1 to 0.8 U/μL GOx concentrations. Measurements were acquired in 100 μM α-ketoglutarate +1X PBS with a 200-rpm stirring rate at 37 °C. Amperometry parameters were +0.7 V vs. Ag/AgCl reference. A representative plot of the response of GABA and GLU (stepwise additions) at the GOx modified Pt UMEs. As expected, no current response to GABA and corresponding stepwise increases in current occurred with increasing GLU concentration. The sensitivity values of the GLU microbiosensors at increasing GOx were 167 ± 35, 199 ± 47, 266 ± 56,335 ± 67, 200 ± 36, 181 ± 29 nA μM^−1^ cm^−2^. Values were shown in mean ± SEM. (one-way ANOVA, *p* < 0.05).

The effect of enzyme concentration on NT sensitivity was carried out using a commercial TRK 8 probe (CenMET, Lexington, KY, USA) with eight Pt microelectrodes [details in 28]. The calibration curves and the sensitivity values for varying GLU and GABA concentrations (20 μM to 60 μM) and GOx enzyme concentrations (0.1 to 0.8 U/μL) is shown in Figure 3. The GLU sensitivity values were in the range of 167 ± 35 to 335 ± 67 nA μM^−1^ cm^−2^. However, the maximum GLU sensitivity was observed for 0.4 U/μL of GOx. This concurs with our previous published work where the authors observed 0.4 U/µL GOx producing the highest GLU sensitivity [31]. Calibration curves at lower GLU concentrations (1 to 40 μM) (Supplementary Information Figure S3) showed sensitivity values in the range of 487 ± 20 to 534 ± 20 nA μM^−1^ cm^−2^ for the biosensors coated with 0.4 U/µL GOx solution. Thus, the best GOx concentration for GLU detection is 0.4 U/µL GOx. Based on Michaelis-Menten theory, one knows the reaction rate (i.e., sensitivity) depends on enzyme concentration. The signal is maximized at the transition point of diffusion limitation and kinetic limitation [22]. It is understandable that for a given enzyme a certain concentration will give the highest sensitivity. These results are comparable to ones published in the literature and thus, are suitable for cell, tissue, and animal model studies [21].

**Figure 4:**
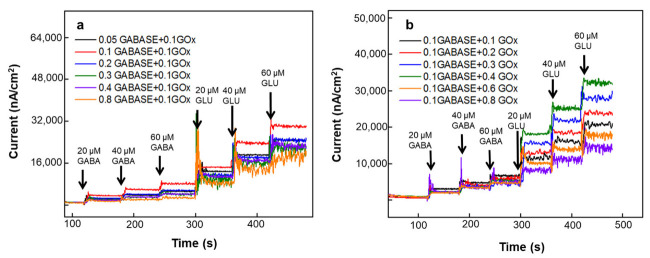
In vitro calibration of GABA microbiosensors at (**a**) different GABASE concentrations (0.05 to 0.8 U/µL) and a fixed GOx concentration (0.1 U/µL) and (**b**) at different GOx concentrations (0.1 to 0.8 U/µL) and a fixed GABASE concentration (0.1 U/µL). The experiments were carried out in 100 μM α-ketoglutarate +1X PBS. The microbiosensors were biased at +0.7 V vs. Ag/AgCl reference. The solution was stirred at 200 rpm and maintained at 37 °C.

Next, the authors studied the effect of GABASE and GOx concentrations on GABA sensitivity. For these experiments different GOx (0.1 to 0.8 U/μL) and GABASE (0.05 to 0.8 U/μL) concentrations with certain combinations (ratios) of these two enzymes were used. For the first set of experiments, the authors observed the GLU and GABA responses with increasing GABASE concentrations (0.05 to 0.8 U/μL) and fixed GOx concentration at 0.1 U/μL (Figure 4a). The GLU biosensor had the same fixed GOx concentration, i.e., 0.1 U/μL. The GABA response was highest at 0.1 U/μL GABASE concentration (red curve). Meanwhile, the GLU responses remain very similar in both GLU and GABA microbiosensors. Also, when GABASE concentration increased (e.g., 0.8 U/µL GABASE + 0.1 U/µL GOx), the signal quality deteriorated. Next, authors systematically varied GOx concentration (0.1 to 0.8 U/μL) and fixed GABASE concentration at 0.1 U/µL (Figure 4b), where the GABA sensitivity was the highest from the above studies. In this case, the maximum GABA sensitivity was observed at 0.1 U/µL GABASE + 0.1 U/µL GOx (black curve). So, the best enzyme combination for GABA detection is 0.1 U/µL GABASE + 0.1 U/µL GOx. However, the maximum GLU sensitivity was observed at 0.4 U/μL. Thus, the best GOx concentration for GLU detection is 0.4 U/µL GOx and the best GABASE concentration for GABA detection is 0.1 U/μL. Higher enzyme concentrations beyond the optimal value leads to low solubility and high viscosity [35], which makes the coating and spotting processes very challenging. Also, this leads to a thicker enzyme layer and thus limits the analyte diffusion across the enzyme layer before it reaches the Pt MEAs [36]. Authors also noted noisy current signals at higher enzyme concentrations when analyte transport becomes diffusion limited. Researchers have used 0.0125 U/μL of GABASE onto similar electrode geometry [36] and achieved sensitivity of about 40 ± 8 nA μM^−1^ cm^−2^. Here, the authors demonstrated GABA sensitivity as high as 141 ± 11 nA μM^−1^ cm^−2^. For the proposed in vivo work, the authors employed 0.1 U/µL GABASE + 0.1 U/µL GOx for the GABA microbiosensor and 0.1 U/µL GOx for the GLU microbiosensor. This is due to the fact that it showed the highest GABA sensitivity with an acceptable GLU sensitivity. The measured GLU and GABA sensitivity values were 278 ± 9 and 21.6 ± 6 nA μM^−1^ cm^−2^ (*n* = 3), respectively.

#### 3.1.3. Effect of mPD Coating Conditions on NT Selectivity

After the immobilization of the enzyme layer, the size-exclusion mPD layer was electrochemically coated onto the Pt UMEs. mPD is a proven coating to achieve selectivity, i.e., the ability to screen out interferents reaching the sensor surface. The calibration curves were generated by using 1 μM to 40 μM range for both GLU and GABA. Calibration was performed by collecting the baseline currents for 10 min, which was followed by stepwise injections of GABA and then GLU with a final injection of 50 µM AA. Among the different mPD coating conditions, 50 mV/s scan rate for 5 cycles showed the maximum GLU and GABA sensitivity combined with a high selectivity. Excellent selectivity towards the major interferent AA was observed. Adequate selectivity towards other common interferents (Supplementary Information Figure S5) such as acetylcholine, choline, dopamine, serotonin, and uric acid was demonstrated using the mPD coating. For further in vitro and in vivo studies, the authors employed these optimal coating parameters. After the mPD coating, the freshly prepared microbiosensors were stored at 4 °C for 24 h and then calibrated and used.

**Figure 5:**
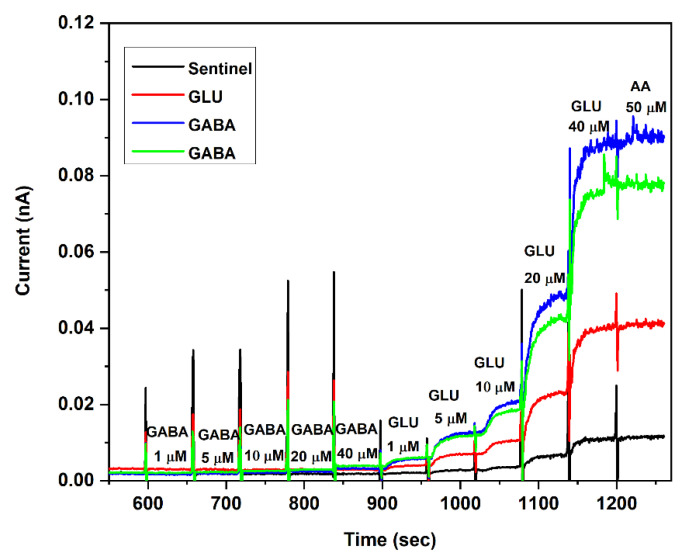
In vitro calibration of GLU and GABA microbiosensors for sensitivity and selectivity measurements. The GOx and GABASE concentrations were 0.1 U/μL each, respectively. The mPD is coated at the optimal conditions. The experiments were carried out in 100 μM α-ketoglutarate +1X PBS. The microbiosensors were biased at + 0.7 V vs. Ag/AgCl reference. The solution was stirred at 200 rpm and maintained at 37 °C.

Figure 5 shows the calibration of GLU and GABA sites with a sentinel site. Further improvements in the selectivity was achieved through subtraction of the interferent signal from the sentinel site [29]. Selectivity was calculated as the sensitivity ratio of GLU or GABA to AA. The GLU sites showed adequate sensitivities, which was comparable to our previous studies [31]. The GLU and GABA sensitivity was 219 ± 8 and 10 ± 1 nA μM^−1^ cm^−2^ (*n* = 3), respectively. The limit of detection (LOD) of the biosensors were as low as 0.11 µM and 5.3 µM for GLU and GABA, respectively (Supplementary Information Table S1).

### 3.2. ODIC Calibration

Authors evaluated the 10 μm diameter microfluidic channel at different flow rates (up to 10 μL/min) for its general characteristics. Amperometry calibration was carried out using 2 μM H_2_O_2_ with the flow rate of 5 μL/min for 540 s (Supplementary Information Figure S6). The oxidation current increased as expected to 0.7 nA for a final concentration of ~18 μM. This corresponds to sensitivity values of 3833 ± 92 to 4110 ± 95 μM^−1^ cm^−2^, which is comparable to the values obtained from similar in vitro calibration experiments. Likewise, the in-situ GLU calibration (2 μM GLU additions for every 5 μL addition with a final concentration of 16 μM) raised the GLU current to ~0.85 nA with sensitivity values ranging between 4300 ± 258 to 6780 ± 352 μM^−1^ cm^−2^. When the flow was stopped, as expected, the GLU current dropped to baseline values within 20 s. The results demonstrate the ability of the ODIC to calibrate NTs in-situ, on demand.

### 3.3. In Vivo Testing of Si Probe

**Figure 6:**
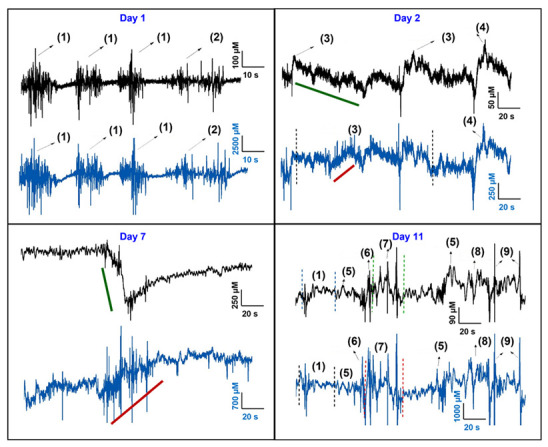
Simultaneous GLU (black curve) and GABA (blue curve) in vivo recordings at different days in Rat 1. The number legends represent: (1) walking, (2) sniffing, (3) Grinding Teeth, (4) moving, (5) external sound, (6) head flick, (7) climbing, (8) head movement, (9) body flick. Day 1 is the day of surgery. The chemical changes are correlated to the rat behavior. Amperometry: +0.7 V vs. Ag/AgCl wire.

Authors measured the changes in the baseline levels of GLU and GABA in the hippocampus of adult male Sprague -Dawley rats. Authors also evaluated the longevity of the biosensors for 11 days. Each recording was acquired for a duration of 1 h. The rats were free to move in their cages during recording periods. The probe was successfully validated by measuring the physiological levels of GLU and GABA with sub-second temporal resolution (~100 ms rise time) in the rat brain in real time (Figure 6). Multiple in vivo recordings were carried out from the day of surgery through 11 days, and fluctuating levels of GLU and GABA were correlated to natural behaviors. For Rat 1, the four Pt UMEs in the probe were coated with the following surface modifications: Site 1 − no enzyme, i.e., sentinel, Site 2 − GOx only, Sites 3 and 4 − GOx + GABASE. The GLU biosensor (site 2) was coated with 0.1 U/μL GOx and the GABA biosensors (sites 3,4) were coated with 0.1 U/μL GOx + 0.1 U/μL GABASE. The probe showed excellent GLU sensitivities, 126 ± 5 and 161 ± 6 nA μM^−1^ cm^2^ (*n* = 3), respectively, during the pre-calibration in vitro. GABA sensitivity was in the range of 5 ± 2 nA μM^−1^ cm^−2^ (*n* = 3).

Numerous data sets were selected for different rat behaviors (events) and analyzed in concentration (μM) vs. time (s). Time frames ranged from 150 s to 1000 s based on capturing selected behaviors such as sniffing, walking, grinding teeth, climbing, resting, rapid movements due to external sounds, and reactions to close objects such as the commutator. In these studies, the periods of rest could be considered as the negative control. There was a clear difference in the peak amplitude of the sensor fluctuations between rest (low) and activity (higher), such as walking and grooming (movement) or when the rat was surprised (a reaction with no movement).

Day 1 recordings showed strong GLU and GABA signals with concentrations changing at the levels of 100′s and 1000′s of μM and with periodic bursts or fluctuations during the “walking” and “sniffing” events. A very similar trend in the changes to the GLU and GABA levels for the “walking” event was observed. Each neuron releases its NT in a series of bursts, each lasting a very short time. This process can repeat over time, and it can happen more or less frequently depending on the brain region and what the rat is doing. This results in fluctuations in the level of neurotransmitter. Each biosensor detects the release of its target NT from many surrounding neurons. When more neurons release NT at the same time, there is a larger concentration than at other times. In addition, there is a biochemical pathway that collects the NTs and recycles them. This happens quickly each time NT is released, and it results in a reduction of NT concentration. Thus, the detected amount of NT fluctuates over time and is related to what is being processed in the brain where the probe is implanted. Day 2 recordings showed a sizeable drop in the GLU and GABA signal intensities and the GLU and GABA concentrations changed at the levels of 10′s and 100′s of μM for “teeth grinding” and “walking” events. The higher values for Day 1 were likely due to release of the NTs from damaged neurons and excitotoxicity from an acute injury response. Some of this difference could also be due to surface fouling. The main reason is that the initial inflammation and macrophage growth during implantation and surgery is expected to cover the sensor sites. For the first “teeth grinding” event, the GLU concentration decreased (green line bar) while the GABA concentration initially did not change but later showed an increasing trend (brown line bar). Day 4 to 11 recordings did not show any further drop in GLU and GABA signal intensities for the same behaviors. One of the events on the day 7 recording showed a quite useful insight about the rat’s behavior. When the rat suddenly noticed the dangling recording cable in proximity, the GLU levels decreased precipitously (green line bar) while the GABA level (brown line bar) steadily increased during the same time and even beyond. This suggests that the rat had more than a momentary reaction to encountering the unexpected object. Day 11 recordings demonstrate the probe was still functional and was able to record the GLU and GABA changes from the various events. Avery similar trend in the changes to the NT levels in other rats was observed.

In another rat (Rat 2), GLU levels from GLU (2 sites) and GABA (1 site) in the probe for 5 days (3 recordings) were recorded. A sample Day 1 GLU recording is shown in Supplementary Information Figure S7. Again, strong GLU signals with concentrations changing at the levels of 10′s of μM and with periodic bursts or fluctuations during the “walking” and “sniffing” events were seen. A very similar trend in the changes to the GLU levels in Rat 1 was observed.

In summary, the authors evaluated and validated the Si probes in 4 rats (4.5 recordings per rat). The results suggest the Si probe could be used to reliably detect concentrations of GLU and GABA in real-time at neural circuit levels in rat models of network function on sub-second timescales over a period of 11 days.

## 4. Conclusions

Implantable silicon probes with four 25-µm-diameter Pt UMEs were optimized in terms of the enzyme loading and non-selective coating methods and validated for simultaneous detection of GLU and GABA in live rats. Calibration of the microfluidic channel demonstrated the probe’s ability to carry out in situ on demand calibration and inject drugs and/or NTs to manipulate neural circuit modulation. Demonstrated feasibility for measuring physiologically relevant changes in the levels of GLU and GABA in real time in freely moving rats for up to 11 days and with no need for external substrates. Eventually, we expect to produce a commercial-ready probe that, for the first time, will enable neuroscientists to better understand the combined role of GLU and GABA in disease and injury, chronically and in real-time, in the live rodent brain.

## Data Availability

Not applicable.

## Supplementary Materials

The following are available online at https://www.mdpi.com/article/10.3390/mi13071008/s1, Principle of GLU and GABA biosensing mechanism, Figure S1: Schematic describing the pathways for GLU and GABA biosensing mechanism, Figure S2: Microspotting process development, Figure S3: In vitro GLU microbiosensor calibration plots, Figure S4: Calibrations curves of GLU, GABA and AA detection with varying mPD coating conditions, Figure S5: Selectivity towards common interferents, Figure S6: Demonstration of ODIC capability and Figure S7: GLU in vivo recordings in Day 1, the day of surgery in Rat, Table S1: LOD of biosensors.
