# Supplementary material for: Brain-Implantable Multifunctional Probe for Simultaneous Detection of Glutamate and GABA Neurotransmitters: Optimization and In Vivo Studies
Source: Micromachines (Basel). 2022 Jun 26;13(7):1008. doi: 10.3390/mi13071008 (PMC9316119; doi:10.3390/mi13071008)
Supplement: Supplementary file 1 [file micromachines-13-01008-s001.zip › micromachines-1763648-supplementary.pdf]

Supplementary Information: Brain-Implantable Multifunctional Probe for Simultaneous Detection of Glutamate and GABA Neurotransmitters: Optimization and In Vivo Studies

Sanjeev Billa [1], Yaswanthi Yanamadala [2], Imran Hossain [1], Shabnam Siddiqui [3], Nicolaie Moldovan [4], Teresa A. Murray[2], and Prabhu U. Arumugam [1,2,*]

[1] Institute for Micromanufacturing (IfM), Louisiana Tech University, Ruston, LA 71272, USA; sbi005@latech.edu (S.B.); buet.imran@gmail.com (I.H.)
[2] Center for Biomedical Engineering and Rehabilitation Science (CBERS), Louisiana Tech University, Ruston, LA 71272, USA; yya012@latech.edu (Y.Y.); tmurray@latech.edu (T.A.M.)
[3] Department of Chemistry and Physics, Louisiana State University Shreveport, Shreveport, LA 71115, USA; shabnam.siddiqui@lsus.edu
[4] Alcorix Co., Plainfield, IL 60544, USA; moldovan@alcorix.com
* Correspondence: parumug@latech.edu; Tel: +(318)-257-5122

Principle of GLU and GABA biosensing mechanism.

At the GLU microbiosensor, glutamate oxidase (GOx) facilitated the breakdown of non-electrically active GLU into α-ketoglutarate (α-keto), NH_3 and H_2O_2 (Equation (S1). A +0.7 V bias on the underlying Pt UME oxidized H_2O_2 molecules, releasing two electrons per H_2O_2 molecule that induced proportional and measurable current. This current was converted into a GLU concentration based on pre-calibration values. The +0.7 V potential helps to decrease noise by reducing interference from interferent molecules that oxidize at higher potentials compared to H_2O_2.

GABA detection is typically facilitated by the application of the GABASE enzyme coating to the Pt UME, but an exogenous application of α-keto was previously necessary to facilitate the breakdown of GABA to produce H_2O_2 (Equations (S2) and (S3)). While this is not an issue for *in vitro* studies, exogenous application of α-keto is easily possible for *in vivo* studies. However, the product of the GOx reaction with GLU is α-keto (Equations (S1) and (S3)). To create the GABA microbiosensor, we applied both GABASE and GOx to the Pt UME to record the combined current generated from GLU and GABA. We then subtracted the current from an adjacent GOx-coated Pt UME to derive the GABA current. The GOx enzyme produced enough α-keto at the GABA microbiosensor to support the conversion of GABA to H_2O_2 by GABASE.

$$GLU_{(E)} + H_2O + O_2 \rightarrow \alpha\text{-ketoglutarate} + NH_3 + H_2O_{2(E)} \quad (S1)$$
$$GABA + \alpha\text{-ketoglutarate} \rightarrow SSA + GLU_{(GABA)} \quad (S2)$$
$$GLU_{(GABA)} + H_2O + O_2 \rightarrow \alpha\text{-ketoglutarate} + NH_3 + H_2O_{2(GABA)} \quad (S3)$$

Abbreviations are, as follow: $GLU_{(E)}$= environmental GLU, $H_2O_{2(E)}$ = H_2O_2 from GLU_E, SSA = succinic semialdehyde, $GLU_{(GABA)}$ = GLU from GABA oxidation, $H_2O_{2(GABA)}$ = H_2O_2 from GABA two-step reaction.

A third probe site (sentinel) was coated identically to the other two sites except that no enzymes were used; this provided a means to measure and subtract signals from electrically active interferent molecules, such as ascorbic acid (AA). An additional size-exclusion layer of m-phenylenediamine (mPD), that rejects larger sized molecules, including most AA molecules, was applied over all the coated probe sites to mitigate the effect of interferents.

Figure. S1 also shows the schematic describing the pathways for GLU and GABA biosensing mechanism.

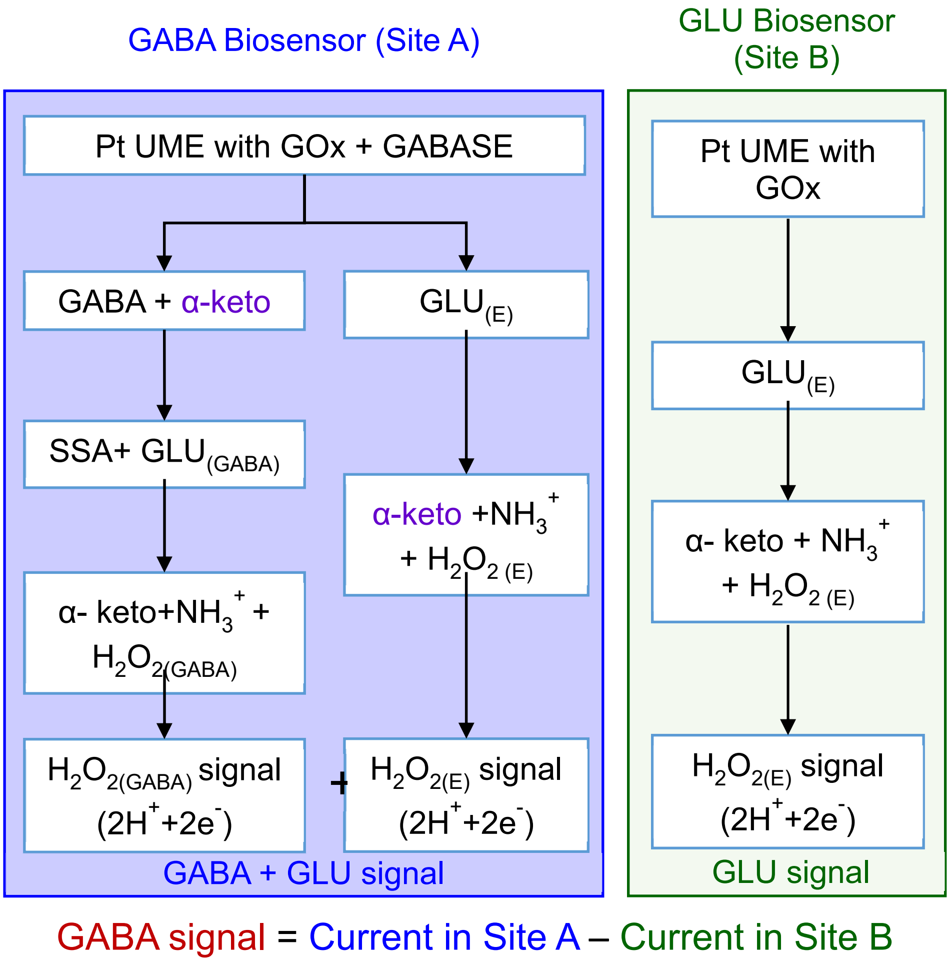

Figure S1. Schematic of the reaction pathways in (**A**) GLU microbiosensor and (**B**) GABA microbiosensor.

Conversion of peak biosensor current responses to peak GLU and GABA concentrations

Calibration curves were created for each GLU and GABA biosensor sites (Figures 6 and 7 in the main text). The conversion of peak current to concentration began with the subtraction of the peak sentinel current ($I_{Sentinel}$) from the raw peak current at the GLU site ($I_{RAW.GLU}$) to derive the peak GLU current (I_{GLU}) as shown in Equation (S4). The GLU current (I_{GLU} in pA) was then divided by the sensitivity of the GLU biosensor (SS_{GLU} in pA/μM) to produce the peak GLU concentration ([GLU] in μM, Equation (S5).

$$I_{GLU} = I_{RAW.GLU} - I_{Sentinel} \quad (S4)$$

$$[GLU] = I_{GLU} / SS_{GLU} \quad (S5)$$

The GABA site detects both GABA and GLU. The current contributed by GLU in the GABA site ($I_{GLU.in.GABA}$, Equation (S6) is the peak concentration of the GLU site ([GLU] in μM) multiplied times the sensitivity of GLU in the GABA site when GABA concentration is 0 μM ($SS_{GLU.in.GABA}$ in pA/μM). To determine the peak GABA current (I_{GABA}), the current contributed by GLU ($I_{GLU.in.GABA}$) was subtracted from the raw GABA site current ($I_{GABA+GLU}$) and the peak sentinel current was also subtracted (Equation (S7). The plot of I_{GABA} versus GLU concentration (not shown here) is used to determine the concentration of GABA. The sentinel is optional if we need to enhance the selectivity values further.

$$I_{GLU.in.GABA} = [GLU] \times SS_{GLU.in.GABA} \quad (S6)$$

$$I_{GABA} = (I_{GABA+GLU} - I_{GLU.in.GABA}) - I_{Sentinel} \quad (S7)$$

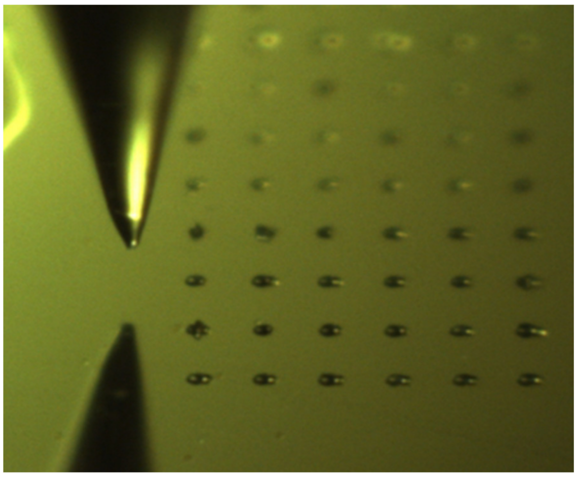

Figure S2. Microspotting process development for simultaneous GLU-GABA detection using the Si probe. Spot size optimization studies by controlling the applied voltage, spotting time and dispenser size.

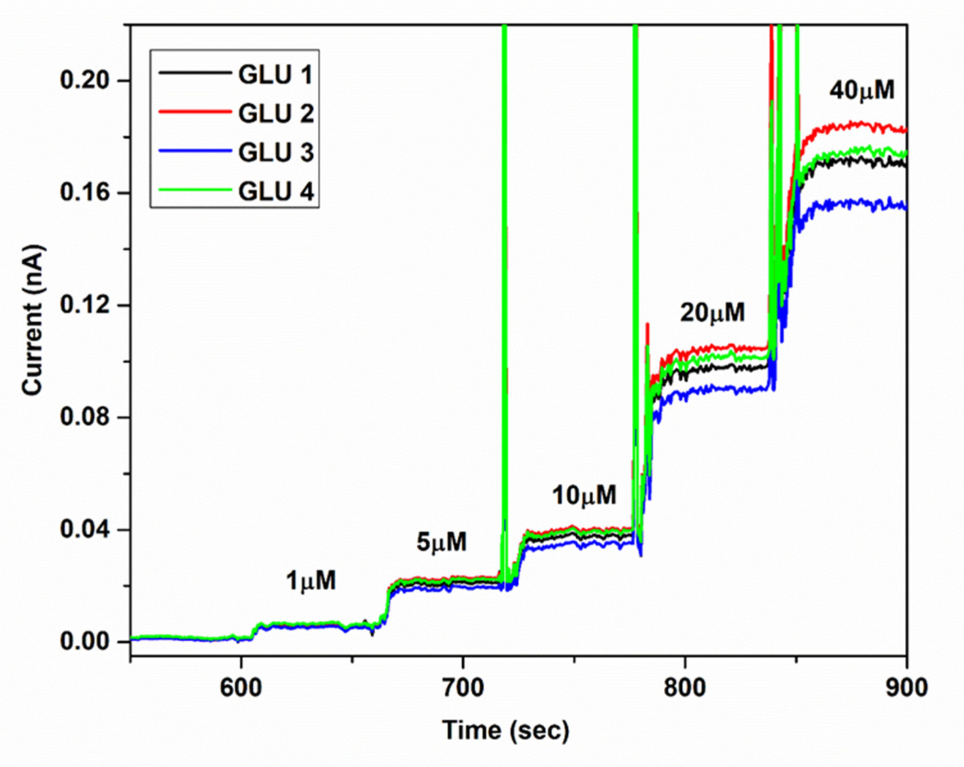

Figure S3. *In vitro* GLU microbiosensor calibration plots. The currents in response to varying GLU concentrations (1 to 40 μM) at the GLU sites 1 to 4 modified with 0.4 U/μL GOx. The sensitivity of GLU sites 1 to 4 are 508±18, 534±20, 487±20, 523±21 nA/μM.cm^2. Values are shown in mean±SEM. The H_2O_2 sensitivity among the UMEs is 5978±87, 6084±84, 5970±75, 6098±83, nA/μM.cm^2 (one-way ANOVA, $p<0.05$). The calibration was done in 1X PBS at room temperature with the solution stirred at 200 rpm. Chronoamperometry was done at +0.7 V vs. Ag/AgCl reference.

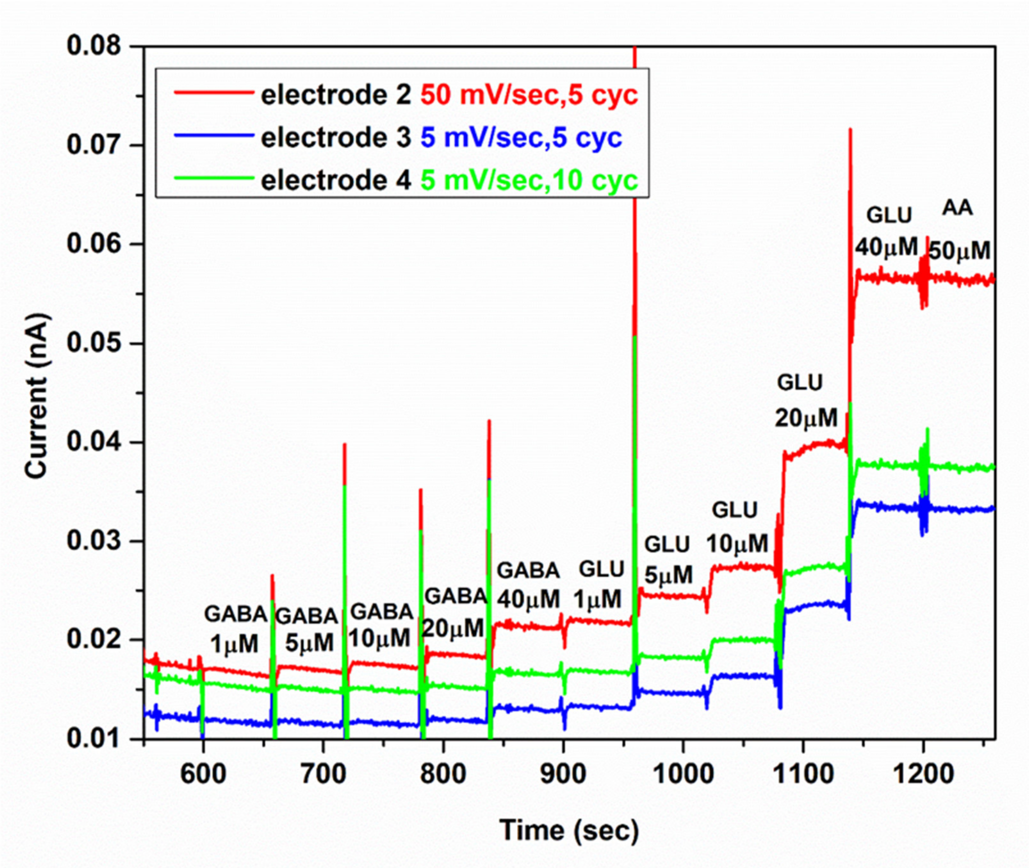

Figure S4. Calibrations curves of GLU, GABA and AA detection with varying mPD coating conditions. Amperometry: + 0.7 V vs Ag/AgCl wire in a stirred 1X PBS beaker, the stir rate is 250 rpm.

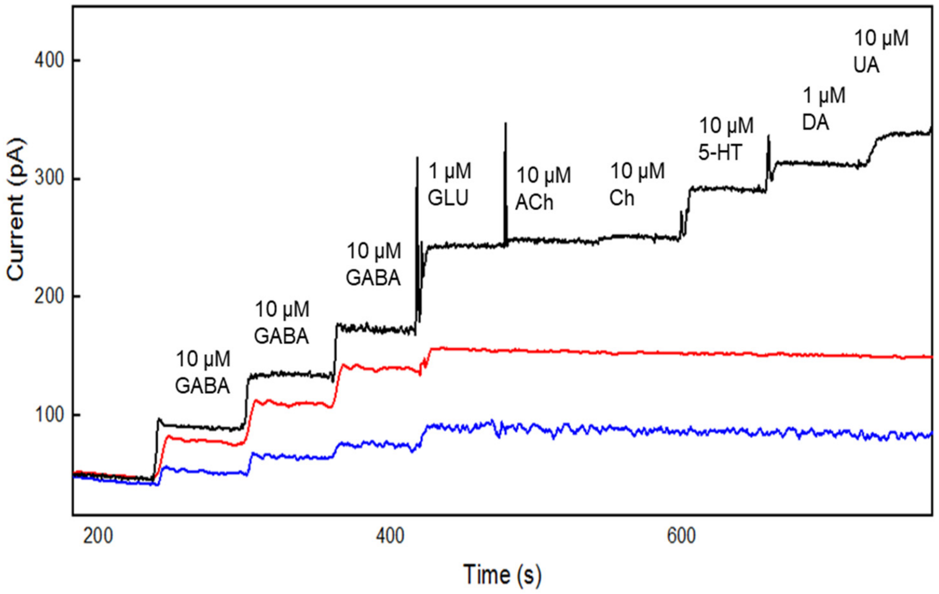

Figure S5. Selectivity towards common interferents such as acetylcholine (Ach), choline (Ch), , serotonin (5-HT), dopamine (DA) and uric acid (UA). No mPD (black curve) and mPD coated at 5 mV/s (blue curve) and 50 mV/s (red curve) scan rates. Amperometry: + 0.7 V vs Ag/AgCl wire in a stirred 1X PBS beaker, the stir rate is 250 rpm.

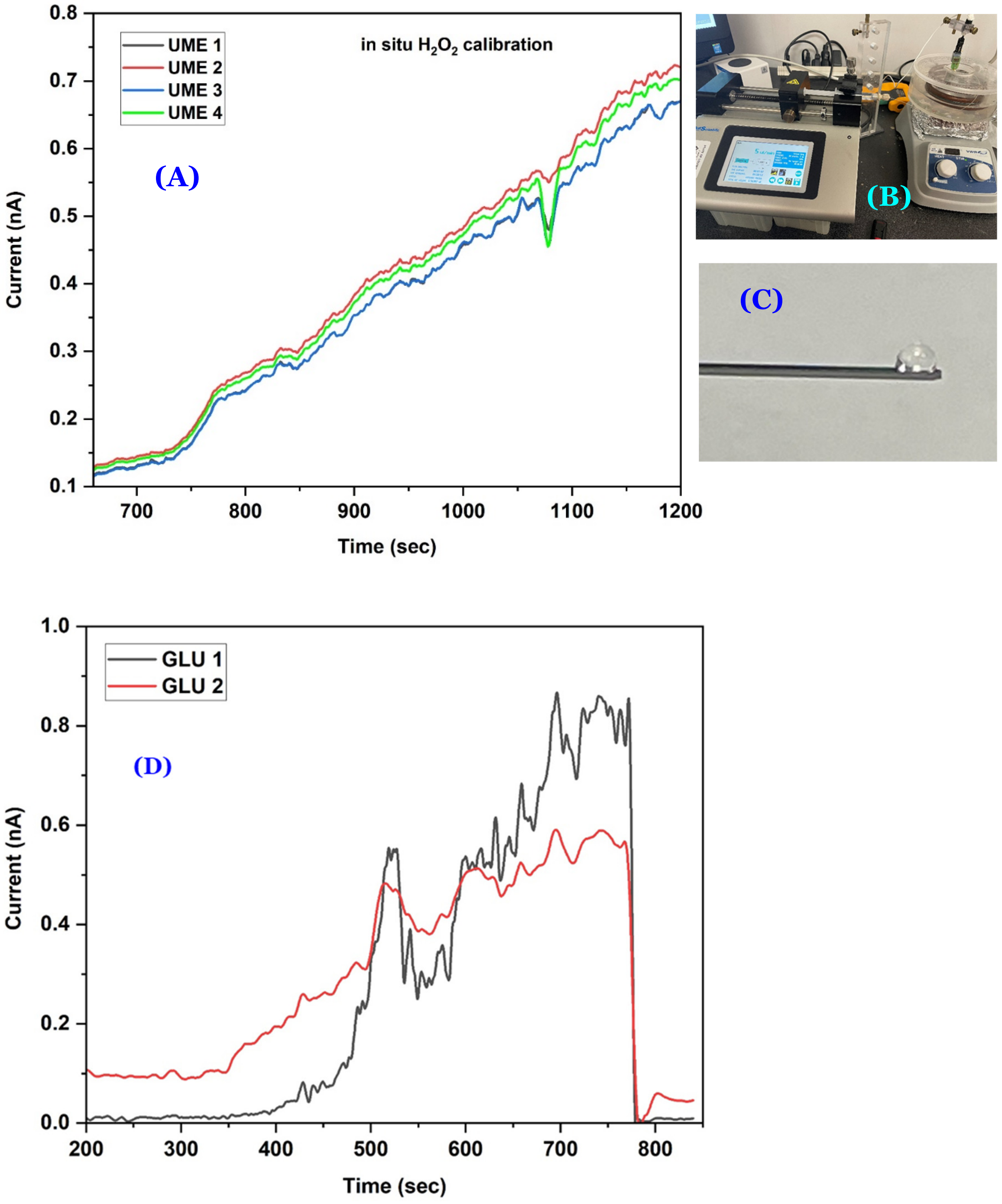

Figure S6. Demonstration of ODIC capability. (**A-C**) H_2O_2 was pumped continuously through the microchannel. Amperometry: + 0.7 V vs Ag/AgCl wire in a stirred 1X PBS beaker. The stir rate is 250 rpm. (**D**) GLU calibration using ODIC capability. GLU was pumped continuously through the microchannel. Amperometry: + 0.7 V vs Ag/AgCl wire in an unstirred 1X PBS beaker.

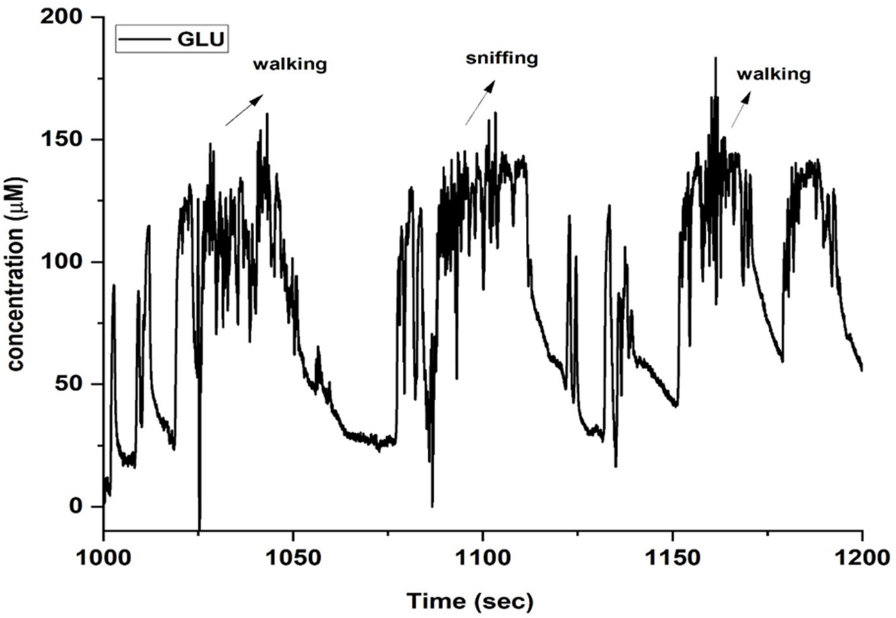

Figure S7. GLU *in vivo* recordings in Day 1, the day of surgery in Rat 2. The chemical changes are correlated to the rat's behavior. Amperometry: + 0.7 V vs Ag/AgCl wire.

Table S1. LOD of biosensors. Values are shown in mean±SEM. The LOD values between Pt UMEs does not vary significantly (ANOVA one-way $p<0.05$).

LOD for GLU sensing

	Sensitivity (nA/μM.cm$^{2)}$	LOD (μM)
GLU	95±3	0.26
GLU in GABA	219±8	0.11
GLU in GABA	179±6	0.12

LOD for GABA sensing

	Sensitivity (nA/μM.cm$^{2)}$	LOD (μM)
GABA	5±0.3	8.6
GABA	10±1	5.3
